# Segmentation and Classification in Digital Pathology for Glioma Research: Challenges and Deep Learning Approaches

**DOI:** 10.3389/fnins.2020.00027

**Published:** 2020-02-21

**Authors:** Tahsin Kurc, Spyridon Bakas, Xuhua Ren, Aditya Bagari, Alexandre Momeni, Yue Huang, Lichi Zhang, Ashish Kumar, Marc Thibault, Qi Qi, Qian Wang, Avinash Kori, Olivier Gevaert, Yunlong Zhang, Dinggang Shen, Mahendra Khened, Xinghao Ding, Ganapathy Krishnamurthi, Jayashree Kalpathy-Cramer, James Davis, Tianhao Zhao, Rajarsi Gupta, Joel Saltz, Keyvan Farahani

**Affiliations:** ^1^Department of Biomedical Informatics, Stony Brook University, Stony Brook, NY, United States; ^2^Center for Biomedical Image Computing and Analytics, University of Pennsylvania, Philadelphia, PA, United States; ^3^Department of Radiology, Perelman School of Medicine, University of Pennsylvania, Philadelphia, PA, United States; ^4^Department of Pathology and Laboratory Medicine, Perelman School of Medicine, University of Pennsylvania, Philadelphia, PA, United States; ^5^Institute for Medical Imaging Technology, School of Biomedical Engineering, Shanghai Jiao Tong University, Shanghai, China; ^6^Department of Engineering Design, Indian Institute of Technology Madras, Chennai, India; ^7^Department of Medicine and Biomedical Data Science, Stanford University, Stanford, CA, United States; ^8^School of Informatics, Xiamen University, Xiamen, China; ^9^Department of Radiology and BRIC, The University of North Carolina at Chapel Hill, Chapel Hill, NC, United States; ^10^Department of Brain and Cognitive Engineering, Korea University, Seoul, South Korea; ^11^Department of Radiology, Massachusetts General Hospital, Harvard Medical School, Boston, MA, United States; ^12^Department of Pathology, Stony Brook University, Stony Brook, NY, United States; ^13^Cancer Imaging Program, National Cancer Institute, National Institutes of Health, Bethesda, MD, United States

**Keywords:** digital pathology, radiology, segmentation, classification, image analysis, deep learning

## Abstract

Biomedical imaging Is an important source of information in cancer research. Characterizations of cancer morphology at onset, progression, and in response to treatment provide complementary information to that gleaned from genomics and clinical data. Accurate extraction and classification of both visual and latent image features Is an increasingly complex challenge due to the increased complexity and resolution of biomedical image data. In this paper, we present four deep learning-based image analysis methods from the Computational Precision Medicine (CPM) satellite event of the 21st International Medical Image Computing and Computer Assisted Intervention (MICCAI 2018) conference. One method Is a segmentation method designed to segment nuclei in whole slide tissue images (WSIs) of adult diffuse glioma cases. It achieved a Dice similarity coefficient of 0.868 with the CPM challenge datasets. Three methods are classification methods developed to categorize adult diffuse glioma cases into oligodendroglioma and astrocytoma classes using radiographic and histologic image data. These methods achieved accuracy values of 0.75, 0.80, and 0.90, measured as the ratio of the number of correct classifications to the number of total cases, with the challenge datasets. The evaluations of the four methods indicate that (1) carefully constructed deep learning algorithms are able to produce high accuracy in the analysis of biomedical image data and (2) the combination of radiographic with histologic image information improves classification performance.

## Introduction

Cancer is a major life-threatening health problem around the world. More than 1.7 million new cancer cases and over 600,000 cancer deaths are estimated in 2019 in the United States alone (Siegel et al., 2019). Brain cancer is one of the deadliest cancer types with low survival rates among both women and men (Siegel et al., 2016; Yuan et al., 2016). Cancer research relies on accurate and reproducible disease characterizations in order to better understand what triggers cancer and how cancer progresses so that more effective means of evaluating cancer interventions can be developed. This requires assembling observational and experimental data at multiple biological scales and fusing information from multiple data modalities.

Biomedical imaging is one of the crucial data modalities in cancer research. Features gleaned from high-resolution, detailed images play a key role in the development of correlative and predictive representations of cancer morphology. Combined with clinical and genomics data, image features can result in more effective data-driven research and healthcare delivery for cancer patients. Biomedical imaging, hence, has evolved into an indispensable tool for researchers and clinicians to extract, analyze, and interpret the complex landscape of diagnostic and prognostic information and to assess treatment strategies. Radiology and the rapidly growing field of Radiomics provide a means of quantitative study of cancer properties at the macroscopic scale. Radiomics deals with the extraction, analysis, and interpretation of large sets of visual and sub-visual image features for organ-level quantification and classification of tumors (Lambin et al., 2012; Gillies, 2013; Aerts et al., 2014; Parmar et al., 2015; Gillies et al., 2016; Zwanenburg et al., 2016). The histopathologic examination of tissue, on the other hand, reveals the effects of cancer onset and progression at the sub-cellular level (Gurcan et al., 2009; Foran et al., 2011; Kong et al., 2011; Kothari et al., 2013; Griffin and Treanor, 2017; Yonekura et al., 2018). Histopathology has been used as a primary source of information for cancer diagnosis and prognosis. Diagnosis and grading of brain tumors, for example, is traditionally done by a neuropathologist examining stained tissue sections fixed on glass slides under a light microscope. Radiology is a more prevalent imaging modality in research and clinical settings. Advancements in digital microscopes made it possible to capture high-resolution images of whole slide tissue specimens and tissue microarrays, enabling increased use of virtual slides in histopathologic analysis.

In this paper, we present the application of state-of-the-art image analysis methods for segmentation and classification tasks for radiographic and histologic image data. We describe a collection of four deep learning-based methods: one method for the segmentation of nuclei and three methods for the classification of brain tumor cases. These methods are from the challenge teams who achieved the top scores at the Computational Precision Medicine (CPM) satellite event of the 21st International Conference on Medical Image Computing and Computer Assisted Intervention (MICCAI2018) and agreed to contribute to this summary manuscript. The CPM event was organized by a subset of the co-authors on this paper as a cluster of image analysis challenges. It is one of the series of challenges organized since 2014 to provide a platform for biomedical imaging research teams to evaluate state-of-the-art algorithms in a controlled environment.

The 2018 CPM event targeted brain diffuse glioma and consisted of two sub-challenges. The *first sub-challenge* was designed to evaluate the performance of algorithms for the detection and segmentation of nuclear material in tissue images. We describe a nucleus segmentation method from this sub-challenge. The method employs an adaptation of the Mask-RCNN algorithm to solve the problem of cell segmentation in hematoxylin and eosin (H&E) stained tissue microscopy images. The authors of this method developed pre- and post-processing steps to further improve the performance of the algorithm. The method achieved a Dice similarity coefficient score of 0.868 when evaluated against a set of manually segmented tissue images. The *second sub-challenge* asked participants to classify lower grade glioma (LGG) cases into oligodendroglioma and astrocytoma subtypes using both radiology and histopathology images. We present three classification methods from this sub-challenge. One of the methods refines lower confidence predictions from a radiology image model by combining predictions from a tissue image model. The second method implements two distinct classification models for radiographic and histologic images and combines them through a dropout-enabled ensemble learning. The third method uses multiple deep learning models: one model for classifying tissue images and two models for segmenting and classifying radiology images. A weighted average operation is then applied to the classification results from tissue and radiology images to assign a class label to each case. The methods achieved accuracy values of 0.90, 0.80, and 0.75, respectively—accuracy was measured as the number of correctly classified cases divided by the total number of cases.

In addition to presenting these algorithms, we intend to make the datasets used in the MICCAI CPM 2018 challenge publicly available to provide a valuable resource for development and refinement of future segmentation and classification algorithms.

## Materials and Methods

In this section, we first present a brief overview of existing work on biomedical image analysis (section “Related Work”). We describe the CPM challenge and datasets in Section “Datasets and Performance Evaluation.” We present the nucleus segmentation method in Section “Instance Segmentation of Nuclei in Brain Tissue Images” and the three classification methods in Section “Methods for Classification of Brain Cancer Cases.”

### Related Work

Computer-aided analysis and interpretation of image data is crucial to maximizing benefits from biomedical imaging. Common image analysis operations include segmentation of regions and objects (e.g., nodules and cells) and classification of image regions and images into categories. Image features and quantitative measures obtained from segmentation and classification can be used in downstream analyses that integrate information from clinical and molecular data and develop predictive and correlative models. Studies have shown the value of image analysis and image features in research, and an increasing number of research projects have developed image analysis methods to efficiently, accurately, and reliably convert raw image data into rich information and new knowledge (Gurcan et al., 2009; Foran et al., 2011; Kong et al., 2011; Kothari et al., 2012, 2013; Lambin et al., 2012; Gillies, 2013; Cheng et al., 2016; Coroller et al., 2016; Gao et al., 2016; Ishikawa et al., 2016; Madabhushi and Lee, 2016; Manivannan et al., 2016; Xing and Yang, 2016; Al-Milaji et al., 2017; Bakas et al., 2017c; Lehrer et al., 2017; Chang et al., 2018a, 2019; Fabelo et al., 2018; Hu et al., 2018; Khosravi et al., 2018; Lee et al., 2018; Mobadersany et al., 2018; Peikari et al., 2018; Saltz et al., 2018; Yonekura et al., 2018; Zhou et al., 2018). Recent work on biomedical image analysis focused on the development and application of machine learning methods, in particular, deep learning models.

The work done by Qian et al. detected and differentiated GBM from solitary brain metastases (van Griethuysen et al., 2017) using a support vector machine (SVM) model. The analysis algorithm computes a variety of radiomic features, using the PyRadiomics package (van Griethuysen et al., 2016; Lu et al., 2019), from contrast-enhanced Radiology image datasets. The experiments show that a combination of the least absolute shrinkage and selection operator (LASSO) and SVM achieves the best prognostic prediction performance and the highest stability. Lu et al. (Krizhevsky et al., 2012) proposed and evaluated an approach, which uses the AlexNet deep learning network (Abrol et al., 2018) as a feature extractor and applies transfer learning to train a model for brain disease detection in magnetic resonance imaging (MRI) data. The last three layers of AlexNet are replaced by a fully connected layer, a softmax layer, and a classification layer to implement the feature extractor function. Chang et al. (2018a) proposed and implemented a CNN model to predict isocitrate dehydrogenase (IDH) mutations in glioma patients using preoperative MRI data. Their experimental evaluation shows that incorporating the age at which a patient was diagnosed with cancer improves algorithm accuracy to 89%. Abrol et al. (Binder et al., 2018) applied feature selection and SVM-based classification methods on MRI data obtained from a group of GBM patients. Their experimental results show that three-dimensional radiomic features computed from radiology images could be used to differentiate pseudo-progression from true cancer progression in GBM patients. Binder et al. (Shukla et al., 2017) identified radiographic signatures of extracellular domain missense mutants (i.e., A289V) of the epidermal growth factor receptor (EGFR) suggestive of an invasive and proliferative phenotype, and associated with shorter patient survival. Their approach leverages the integrated analysis of advanced multi-parametric MRI (Bakas et al., 2016) and biophysical tumor growth modeling (Akbari et al., 2018). Their findings were corroborated by experiments *in vitro* and *in vivo* in animal models, contributing to the discovery of a potential molecular target and presenting an opportunity for potential therapeutic development (Shukla et al., 2017). Another study (Bakas et al., 2017a) found an imaging signature in radiology images of the most prevalent mutation of EGFR, namely, EGFRvIII, revealing a complex yet distinct macroscopic GBM radiographic phenotype. This signature showed a classification accuracy of ∼90% for determining EGFRvIII GBM tumors. The study used an SVM model for multivariate integrative analysis of multiple image features to identify the signature. The features include the tumor’s spatial distribution pattern leveraging a biophysical growth model (Akbari et al., 2018) and a distinct within-patient self-normalized heterogeneity index (Wang et al., 2019).

Mobadersany et al. (2018) examined the application of deep learning techniques to predict outcomes in LGG and glioblastoma multiforme (GBM) patients. Their approach combines tissue image analysis results with genomics data to achieve high accuracy. The deep learning network consists of convolutional layers, which are trained to predict image patterns associated with survival. This network is connected to fully connected layers that transform the image features for survival analysis. Survival data are modeled via a Cox proportional hazard layer. Wang et al. (Qian et al., 2019) implemented an analysis pipeline to classify glioma cases into grades II, III, and IV gliomas using whole slide tissue images (WSIs) from H&E and Ki-67 stained tissue samples. The pipeline consists of multiple steps, including region-of-interest (ROI) identification, image feature extraction, feature selection, automated grading of slides, and interpretation of the grading results. Multiple image features, such as the shapes and sizes of nuclei and image intensity distribution, are computed and pruned using a random forest method. The grading step employs machine learning models with automatic tuning of model parameters for the best classification performance. Saltz et al. (2018) employed a deep learning workflow to create maps of tumor-infiltrating lymphocytes (TILs) in more than 5,000 WSIs from 13 different cancer types in The Cancer Genome Atlas (TCGA) repository. The image analysis approach partitions each WSI into small (50 μm by 50 μm) patches and classifies each patch as either TIL-positive or TIL-negative. The workflow implements an iterative learning phase in which predictions by the deep learning models are reviewed and corrected by pathologists, to refine and improve classification accuracy. The analysis method also uses a convolutional neural network (CNN) to identify and segment regions of necrosis in order to reduce false positives.

Nucleus segmentation is one of the core analysis tasks in histopathology imaging projects which study tissue morphology (Gurcan et al., 2009; Madabhushi and Lee, 2016; Xing and Yang, 2016). The nucleus segmentation task is challenging because of the relatively large variation in the intensity of captured signal and the ambiguity of boundary information when separating neighboring nuclei. Several projects proposed machine learning algorithms that use engineered image features and algorithms that perform statistical analyses of intensity and texture properties to detect and delineate nucleus boundaries (Kong et al., 2011; Gao et al., 2016; Peikari and Martel, 2016; Peikari et al., 2018). In recent years, there has been a significant shift toward the application of deep learning techniques. Yang et al. (2018) proposed a method that uses a U-Net model to segment lesions in cervical cancer cases. The segmentation results are fed into a cascade network, which integrates the foreground and the edges of the segmented nuclei to generate instance segmentations. Wollmann et al. (2019) developed a hyperparameter optimization method that searches for the best parameters of a nucleus segmentation pipeline to improve segmentation accuracy. The authors evaluated their technique with two analysis pipelines, a clustering-based pipeline and a deep learning pipeline, using prostate cancer tissue images. Their results show that the deep learning pipeline performs better than the clustering-based pipeline. Alom et al. (2018) proposed a residual recurrent CNN built on the U-Net architecture (Ronneberger et al., 2015). While this type of network has been used for segmentation of macro-level objects such as retinal blood vessels and the lungs, the authors adopted it to segmentation of the nuclei. Xie and Li (2018) implemented a neural network method that learns object-level and pixel-level information in tissue image patches. The goal is to have the analysis pipeline carry out nucleus detection and nucleus segmentation simultaneously. Hou et al. (2019b) proposed a sparse convolutional autoencoder for the detection of nuclei and feature extraction in WSIs. The approach integrates nucleus detection and feature learning in a single network. The network encodes the nuclei into sparse feature maps, which represent the nuclei’s locations and appearances and can be fine tuned for end-to-end supervised learning.

Radiology and pathology capture morphologic data at different biological scales. The non-invasive and non-ionizing property of MRI made it quite popular for oncology imaging studies such as brain tumors (Bakas et al., 2016). On the other hand, the *de facto* standard for tumor assessment and grading is whole slide tissue biopsy examined under a microscope. Combined use of image modalities from both domains can lead to improvements in image-based analyses. Lundstrom et al. (2017) argue for a tighter collaboration between radiology, pathology, and genomics teams toward enhanced integrated diagnosis of disease. The authors point to the increasing use of digital slide technologies in pathology as well as to the fact that computational approaches for radiology and pathology imaging modalities are not fundamentally different. They note that combining complementary views of the disease from multiple scales can maximize the benefits of biomedical imaging. Madabhushi and Lee (2016) note that researchers are increasingly looking at opportunities for combining radiomic data with features extracted from high-resolution pathology image for better predictive capabilities in disease prognosis. On the methodology and software front, Arnold et al. (2016) developed a web-based platform that integrates radiology and pathology data for cancer diagnosis. Saltz et al. (2017) devised methods and tools for combined computation, management, and exploration of image features from radiology and pathology image datasets. Kelahan et al. (2017) implemented a dashboard for radiologists to view pathology reports to aid with diagnosis and image-guided decision making. McGarry et al. (2018) proposed a method for combining multi-parametric MRI data with digital pathology slides to train predictive models for prostate cancer localization.

Despite a growing body of research and development on methods and tools, computerized image analysis continues to be a challenging task. Both image resolutions and data complexity continue to increase, requiring the enhancement of existing methods and the development of new techniques. For example, contemporary digital microscopy scanners are capable of imaging whole slide tissue specimens at very high resolutions (e.g., over 80,000 × 80,000 pixels). These images may contain millions of cells and nuclei, and multiple types of regions (e.g., tumor, stromal, and normal tissues). There can be significant morphological heterogeneity within a specimen, as well as across specimens in both radiographic and histologic imaging, requiring novel methods that can handle heterogeneity and increasing the density of morphologic information.

### Datasets and Performance Evaluation

The approaches, which will be described in Sections “Instance Segmentation of Nuclei in Brain Tissue Images” and “Methods for Classification of Brain Cancer Cases,” were experimentally evaluated with radiographic and histologic image datasets from the MICCAI 2018 CPM challenge event. Here we provide a brief description of the challenge datasets and the methods for scoring algorithm performance. The datasets for the 2018 CPM challenge were obtained from TCGA^1^ (Tomczak et al., 2015) and The Cancer Imaging Archive (TCIA^2^) (Clark et al., 2013; Prior et al., 2013) repositories, and the images had been scanned at the highest resolution. Images from these sources are publicly available and have been used in many publications (e.g., Aerts et al., 2014; Yu et al., 2016; Bakas et al., 2017c; Mobadersany et al., 2018; Saltz et al., 2018; Agarwal et al., 2019).

#### Datasets for Segmentation of Nuclei in Pathology Images

A WSI may contain hundreds of thousands of nuclei; some images with large tissue coverage will have more than one million nuclei. Manually segmenting all nuclei in the entire WSIs would be infeasible. Thus, we extracted image tiles from WSIs and used the tiles in the training and test datasets in order to reduce the cost of generating high-quality ground truth data as well as the computational requirements of the training and test steps of analysis algorithms. The image tiles were selected by a pathologist and extracted from a set of GBM and LGG WSIs at the highest resolution. The training and test datasets consisted of 15 and 18 image tiles, respectively. The sizes of the tiles ranged from 459 × 392 pixels to 1032 × 808 pixels in the training set and from 378 × 322 pixels to 500 × 500 pixels in the test set. The nuclei in each image tile were segmented by two students. The segmentation results were reviewed, refined, and consolidated by the pathologist to generate the final set of segmentation data. This process generated 2905 and 2235 nuclei in the training and test sets, respectively.

In the challenge event, the performance of a segmentation algorithm was measured as the average of the standard Dice similarity coefficient and a modified version of the Dice metric. The standard Dice score (Dice, 1945) measures the overlap between two sets of segmentation results without taking into account the individual nuclei. That is, it computes the amount overlap between the ground truth mask and the mask generated by the segmentation algorithm without considering splitting and merging of the nuclei by the algorithm. The modified Dice metric aims to incorporate split and merge errors into the score. We refer the reader to an earlier publication (Vu et al., 2019) for a more detailed description of the modified Dice metric.

#### Datasets for Combined Radiology and Pathology Classification

The datasets were matched MRI and digital pathology images obtained from the same patients and the same time point. Each case corresponded to a single patient. There was one set of MRI data (T1, T1C, FLAIR, and T2 images) and one corresponding WSI for each case. The training set contained a total of 32 cases: 16 cases that were classified as oligodendroglioma and 16 cases classified as astrocytoma. The test dataset consisted of 20 cases with 10 cases of oligodendroglioma and 10 cases of astrocytoma. We retrieved the WSI and MRI images from the TCGA and TCIA archives, respectively. These images had been obtained and classified following the protocols implemented in the TCGA project^3^. We obtained the ground truth classification labels of the cases from the associated clinical and metadata in the TCGA repository. These classifications were further reviewed by a pathologist and a radiologist. In the challenge event, we used the accuracy of a classification method to score its performance and rank it. We counted the number of correctly classified cases and divided that number by the total number of cases to compute the accuracy score.

In the following sections, we will present a nucleus segmentation algorithm (section “Instance Segmentation of Nuclei in Brain Tissue Images”), which achieved the second highest score in the segmentation challenge, and three classification algorithms (section “Methods for Classification of Brain Cancer Cases”), which achieved the top three scores in the classification challenge.

### Instance Segmentation of Nuclei in Brain Tissue Images

In this section, we present the nucleus segmentation algorithm developed by XR, QW, LZ, and DS. This method achieved the second highest score in the CPM challenge and its developers agreed to contribute to this manuscript.

**FIGURE 1:**
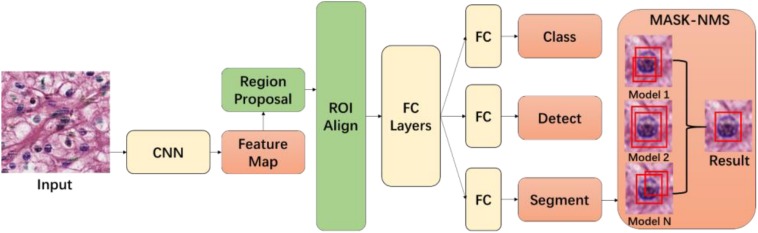
Tissue image segmentation model. The first part of the model consists of the Mask-RCNN module. Output from this module is input to the MASK-NMS module for final segmentation prediction output.

The method implements an application of the Mask-RCNN network (He et al., 2017) with a novel MASK non-maximum suppression (MASK-NMS) module, which can increase the robustness of the model. Mask-RCNN is a deep learning network extended from the Faster-RCNN model (Ren et al., 2015) and is used to carry out semantic and object instance segmentation (see Figure 1). In our implementation, we used ResNet-101 to build a Mask-RCNN pyramid network backbone for the segmentation of nuclei in WSIs. This adaptation is based on an existing implementation by Matterport^4^. We have extended this implementation in several ways to improve segmentation performance. First, we have reduced the region proposal network (RPN) anchor sizes and increased the number of anchors to be used because the nuclei are small objects and can be found anywhere in a tissue image. Second, we have increased the maximum number of predicted objects, since even a small image tile from a tissue slide can contain 1000 or more nuclei. Moreover, rather than training the network end-to-end from the start, we initialized the model using weights from the pre-training on the MSCOCO dataset (Lin et al., 2014). We train the layers in multiple stages. We first train the network heads after they are randomly initialized. We later train the upper layers of the network. After this, we reduce the learning rate by a factor of 10 and train the entire network end to end. In our experiments, the training took 300 epochs using stochastic gradient descent with momentum set to 0.9. During training and testing, input tissue images were cropped to 600 × 600.

In addition to the above extensions, we implemented a set of pre-processing steps to further improve the algorithm performance. Holes in the masks are filled by an image morphology operation. Fused nucleic masks are split by applying morphological erosion and dilation. To help avoid overfitting, data augmentation, which could increase the amount of training data, is applied in the form of random crops, random rotations, Gaussian blurring, and random horizontal and vertical flips.

Our implementation combines predictions from fivefold cross training models in a post-processing step (see Figure 1). We have implemented this step in a novel module called MASK-NMS, which is one of our contributions in the segmentation method. MASK-NMS takes unions of masks with maximum overlap and removes false-positive masks with a small overlap. It starts with a set of segmentation results. This set is called *I*. Each result in set *I* is assigned a score *S*, which is the value of the classification probability from the Mask-RCNN module and corresponds to the confidence level of the segmentation result. After selecting the segmentation with the maximum score *M* (the maximum score among scores *S*), MASK-NMS removes it from the set *I* and appends it to the final segmentation set *D*. *D* is initialized to an empty set. It also removes any segmentations with an overlap greater than a threshold *N* in the set *I*, where the intersection over union (IOU) is used as the overlap metric. IOU is also known as the Jaccard similarity index (Jaccard, 1901), which measures the similarity between finite sample sets. It is defined as the size of the intersection between two sets divided by the size of the union of the sets. The selection process repeats until set *I* becomes empty. Finally, we obtain the segmentation results in set *D*. The MASK-NMS module assembles multiple results together and reduces false positives and false negatives.

### Methods for Classification of Brain Cancer Cases

In this section, we present three classification algorithms, which achieved the top three scores in the classification challenge and the developers of which agreed to contribute to this manuscript.

#### An Approach for Classification of Low-Grade Gliomas Using Combined Radiology and Pathology Image Data

**FIGURE 2:**
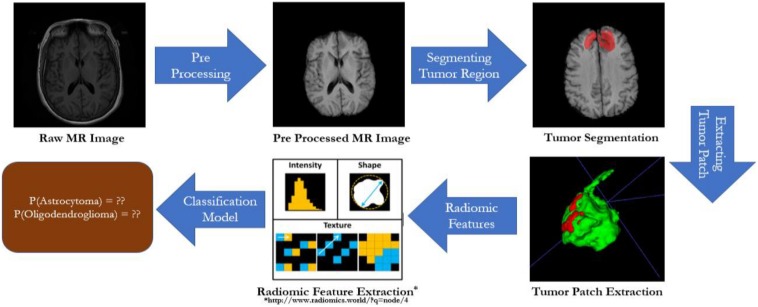
Radiology image analysis. Images are pre-processed (i.e., skull stripping and co-registration) before they are analyzed through the remaining steps of the analysis pipeline. After the pre-processing step, tumor regions in the images are segmented via a CNN model. This step is followed by computation of a set of 105 radiomic features in segmented regions. The high-dimensional feature vector is reduced to a 16-dimensional feature vector using the principle component analysis method. A classification network is trained with these feature vectors.

**FIGURE 3:**
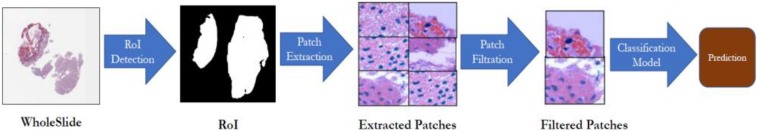
Pathology image analysis. A region-of-interest (ROI) step detects and segments tissue regions. The tissue regions are partitioned into patches. Distinct patches are filtered using the isolation forest technique. The prediction represents the probability values of the case being astrocytoma or oligodendroglioma.

**FIGURE 4:**
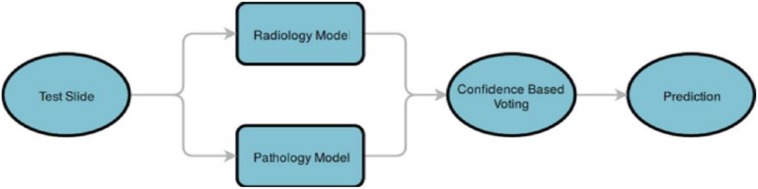
Combining predictions from the pathology and radiology models. A test case is analyzed by the radiology classification model and the pathology classification model. The results from the two models are processed in a confidence-based voting step, which chooses the class with the highest prediction probability value.

The top-performing method (developed by AB, AsK, AvK, MK, and GK) (Bagari et al., 2018) in the classification challenge uses an MRI classification model and a WSI classification model and combines the predictions from the two models to assign a class to a given case. The overall analysis pipeline is depicted in Figures 2–4 and described below.

##### Radiology image analysis pipeline

Different pulse sequences in MRI, including native T1- and T2-weighted, T2-Flair, and T1-weighted post-contrast imaging, can be used to enhance different parts of a tumor. In this part of the pipeline (Figure 2), we execute a segmentation pipeline consisting of the following steps on these images before features are computed from the images and used in the classification model: (1) Skull stripping: It is necessary to remove the skull from MRI as its presence can be wrongly interpreted as a tumor, and most segmentation networks are trained using skull-stripped images. (2) Co-registration and re-sampling to isotropic voxel spacing: Following skull stripping is the step of co-registering the MRI sequences to a reference sequence. Generally, there can be movement between scans if the patient does not remain still or if the scan is acquired on a different day or using a different scanner. Registered images are spatially correlated across channels and can be used for tumor segmentation. We register sequences T1, FLAIR, and T2 with respect to T1c scan. The MRI volumes are re-sampled to an isotropic voxel resolution of 1 mm^3^ after the co-registration step. (3) Segmentation of tumor regions using a CNN: Tumor regions are segmented by a fully CNN trained on the BraTS-2018 dataset (Menze et al., 2014; Bakas et al., 2017b, c, 2018; Crimi et al., 2018). After the segmentation step, a set of 105 radiomic features are computed on segmented regions using the pyradiomic library (Lu et al., 2019). These features include shape features, first-order statistics, features from gray level co-occurrence matrix, features from gray level run length matrix and gray level size zero matrix, and neighboring gray tone difference matrix. The 105-dimensional radiomic feature vectors are reduced to a 16-dimensional feature vector using the principal component analysis. A classification model is trained with 16-dimensional feature vectors as input. If the training dataset has N cases, the model is trained with an (*N*,16) input using logistic regression with the liblinear optimization algorithm (Fan et al., 2008) and a fivefold cross-validation process. This process fits a logistic regression model on the entire training data. Classification predictions from the MRI data are obtained using this model.

##### Analysis pipeline for whole slide tissue images

Tissue slides may contain large areas of glass background that are irrelevant to image analysis and should be removed. In this part of the pipeline (Figure 3), in order to detect and segment tissue regions and remove regions corresponding to glass background, a tissue image is first converted from the RGB color space to the HSV color space. Then, lower and upper thresholds are applied on color intensities to get a binary mask. The binary mask is processed to fill in small holes and remove clustered clumps from foreground pixels. After this step, bounding boxes around all the discrete contours are obtained. The bounding boxes serve as blueprints for the patch extraction process. The patch extraction process partitions the segmented tissue region into 224 × 224-pixel patches. The 224 × 224-pixel patches are color-normalized (Reinhard et al., 2001) and assigned the same label as the label of the WSI. A subset of distinct patches is filtered out using an outlier detection technique called the Isolation Forest (Liu et al., 2008). The filtering step is executed as follows. We train an autoencoder with a pixel-wise reconstruction loss to generate feature vector representations of patches from the input image. The isolation forest method is then executed with these feature vectors to find outlier patches. The remaining patches after the outlier detection step are used to refine a DenseNet-161 network, which has been pre-trained on ImageNet. Binary cross entropy is used as the loss function. During the prediction phase, test patches extracted from a WSI are classified using the trained model, and a probability score is assigned to the image based on a voting of classes predicted for individual patches.

##### Combining predictions

As is shown in Figure 4, finally, predictions from both the radiology and pathology models are compared, and the class label of a case is determined based on the model, which gives a prediction with a higher probability score.

#### Dropout-Enabled Ensemble Learning for Multi-Scale Biomedical Image Classification

This method is the second best performing (developed by AM, MT, and OG) (Momeni et al., 2018) and proposes two distinct classification models for radiographic and histopathologic images and their integration through dropout-enabled ensemble learning.

##### Radiology classification model

**FIGURE 5:**
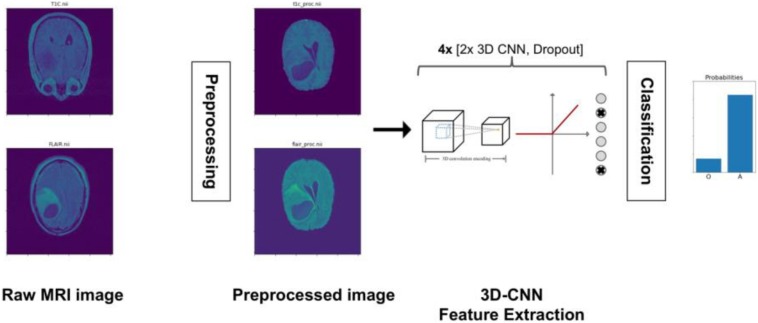
Radiology image analysis pipeline. Radiology images are pre-processed for bias field correction, skull stripping, and co-registration before they are input to a 3D CNN. The 3D CNN is trained to output a prediction (probability) value for each case as to whether the case is oligodendroglioma (O) or astrocytoma (A).

As is shown in Figure 5, radiology images are pre-processed through a pipeline of bias field correction, skull-stripping, and co-registration steps before they are input to a 3D CNN network. The 3D CNN consists of eight layers to extract deep features from MRI and three 3D max pooling to reduce the sample size. The input of the 3D CNN is a 3D voxel image in the form of three spatial dimensions and two modalities per voxel. In this work, the 3D CNN is trained with the T1c and T2-FLAIR modalities only because these are the most informative for LGG segmentation. After the last convolutional layer, the extracted features are averaged over all of the 3D space to yield a unique 100-dimensional feature vector per case. This vector is connected to a 1D output for classification with cross-entropy loss. The whole network is then trained. To avoid overfitting, we use classical data augmentation techniques (rotation, cropping, etc.) as well as dropout. Eight dropout layers are placed throughout the network to avoid overfitting and for the ensemble learning step.

##### Histopathology classification model

**FIGURE 6:**
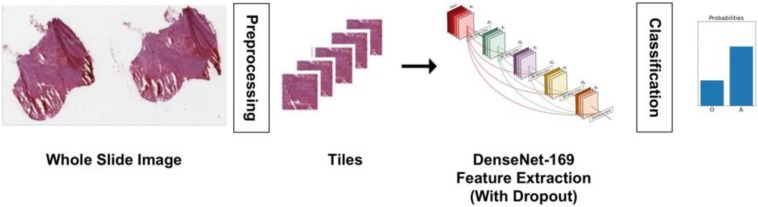
Histopathology image analysis pipeline. The whole slide tissue images are pre-processed to detect tissue, do color normalization, and extract tiles. The tiles are input to a DenseNet model for classification. The model outputs the probability of a case being oligodendroglioma or astrocytoma.

A multiple instance learning approach, as shown in Figure 6, is implemented for the histopathology images. The learning step is carried out after a pre-processing phase. The pre-processing steps here consist of tissue detection, color normalization, and tiling. Tissue detection is done with Otsu thresholding to detect and segment tissue regions only, eliminating regions that are glass background. A simple histogram equalization algorithm is used for color normalization prior to tiling. The tiling step extracts 20 448 × 448-pixel patches from a WSI by uniform random sampling. Once the image patches have been extracted, a DenseNet network pretrained on ImageNet is fine tuned, after removing its last fully connected layer. The remainder of the network is used as a fixed feature extractor for tissue images, and two fully connected layers with dropout are used for classification. As with the radiology model, we used classical data augmentation techniques along with dropout to eliminate overfitting.

##### Ensemble learning model

**FIGURE 7:**
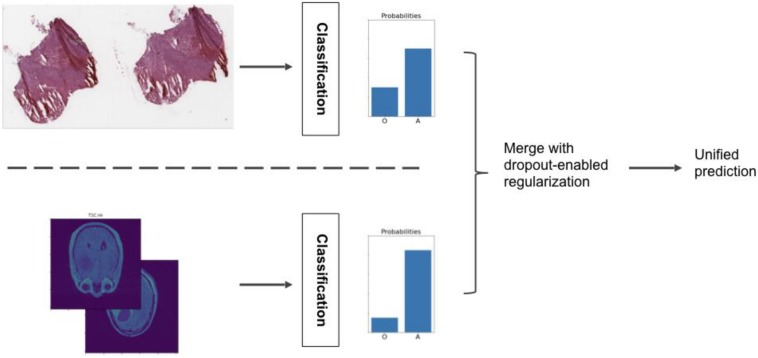
Ensemble model that combines classifications from the radiology and histopathology image analysis pipelines.

The main contribution of our approach is a meta-algorithm that combines the histopathology and radiology classification models, as is shown in Figure 7. In this ensemble learning methodology, each model is trained separately. Their predictions are combined into a single, more robust output. The basic idea is to extract the one-to-last feature layer from each individual classification model and form a single feature vector for each case/patient by concatenating the two feature vectors. An SVM model is then trained with the combined feature vectors to classify the cases. However, if the training dataset is small (which is the case with the CPM challenge dataset; we have 50-dimensional feature vectors from both the classification models and only 32 cases in the training dataset), the classification problem can become under-determined and result in overfitting of the models. To address this problem, we use regularization through dropout in the ensemble learning step. The idea is to enable the dropout values of the models in the test phase, so that individual models produce multiple (typically thousands) feature vectors for each subject. These many feature vectors can then be concatenated to form the combined feature vectors, creating a training dataset big enough for the SVM model (Momeni et al., 2018). Dropout at test time results in sampled feature vectors that are both distinct and informative and provides sufficient variance in the training dataset. Hence, the ensemble learning method can learn a more accurate and robust model from the newly produced dataset.

#### A Weighted Average-Based Classification Method

**FIGURE 8:**
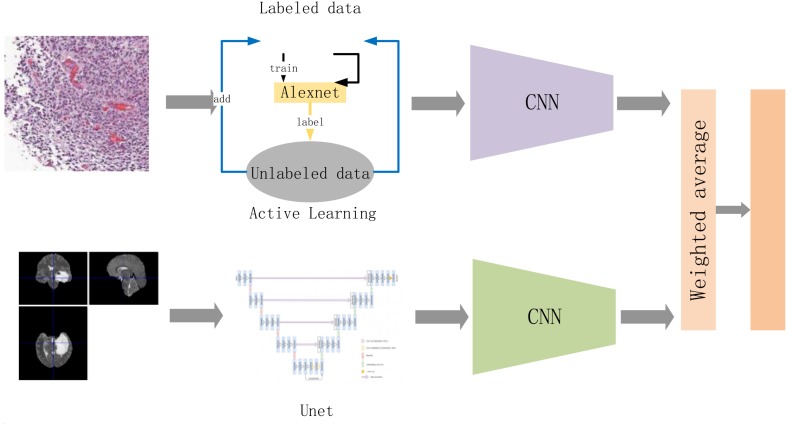
The flow of the entire method. Among them, we slice the entire pathological data and extract the effective diseased area as much as possible. The active learning strategy follows our work in Qi et al. (2018). The goal of that work is to maximize learning accuracy from very limited labeling data. The classification model is updated iteratively with an increasing training set. The sliced pathological data are sent to a convolutional neural network to obtain the discrimination results of the pathological data. The radiological data are sent to the Unet and CNN to obtain classification results after preprocessing. Finally, the results are combined via a weighted average operation to obtain the final result.

The third best performing method (developed by QQ, YZ, YH, and XD) is illustrated in Figure 8. It analyzes each imaging modality (radiology images and pathology images) separately and combines the prediction results via a weighted average operation. We describe the individual classification models and weighted average operation below.

Classification of pathology images is carried out by identifying tissue characteristics that differentiate oligodendroglioma from astrocytoma. Astrocytoma is noted to have more grades, as well as necrosis, increased cell density, calcification, and nuclear atypia. On the other hand, fried egg-like cells, and the tissue characteristics of chicken-cage-like blood vessels are unique to oligodendroglioma. In the proposed method, each histologic image is partitioned into 512 × 512 patches. A sample set is created to identify typical samples of both subtypes of brain diffuse gliomas to assess imbalance in the data. In order to prevent the classification error caused by data imbalance, our method expands the sample set by rotating the original image in symmetrical and asymmetrical directions. The balanced samples are then sent to a CNN classifier network, which is trained to fully recognize the tissue and cell characteristics of oligodendroglioma and astrocytoma (see Figure 8). The method uses the VGG16 CNN network (Simonyan and Zisserman, 2014). We use data augmentation and add dropout layers or batch normalization layers to the classification model to reduce the risk of overfitting the model.

**FIGURE 9:**
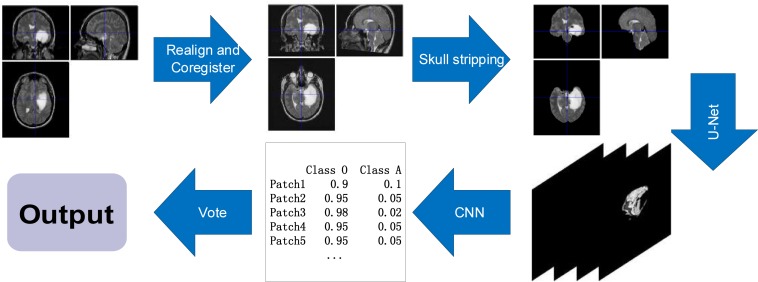
The classification process of radiology images. The process aligns the images of different modalities through realignment and co-register, extracts brain tissue through skull stripping, extracts lesion area by the U-Net, and classifies cases by CNN.

The classification model for radiology images is shown in Figure 9. Radiology images are pre-processed using methods from the SPM12 software (Penny et al., 2011). The methods include *Realign, Estimate, and Re-slice* to register data of the same modality in different cases; *Co-register and Estimate and Re-slice* to register different modal data of the same case; and *Segment and ImCalc* to extract the intracranial cavity. The pre-processed images are then segmented using the U-Net (Ronneberger et al., 2015) segmentation network. Patches with tumor, which are predicted by the segmentation network, are used as training data for a 2D Densenet (Huang et al., 2017) network. We classify each patch, set the threshold value of 0.99, and select effective patches. The ensemble of multiple patches can effectively improve the robustness of the classifier.

Classification results from the radiology image dataset and the pathology image dataset are combined via a weighted average operation (see Figure 8):

$$\hat{y}=\alpha^{*}\,f\,\left(X_{p}\right)+{\left(1-\alpha \right)}^{*}\,g\,\left(X_{r}\right)$$

where the classifiers for pathology data and radiology data, *X*_p_ and *X*_r_ are VGG16 and DenseNet, respectively. *f*(●) and *g*(●) represent the probabilities acquired from softmax function in *X*_p_ and *X*_r_. The weight α is empirically estimated in predicting the final classification label $\hat{\text{y}}$.

## Experimental Results

### Segmentation of Nuclei

**FIGURE 10:**
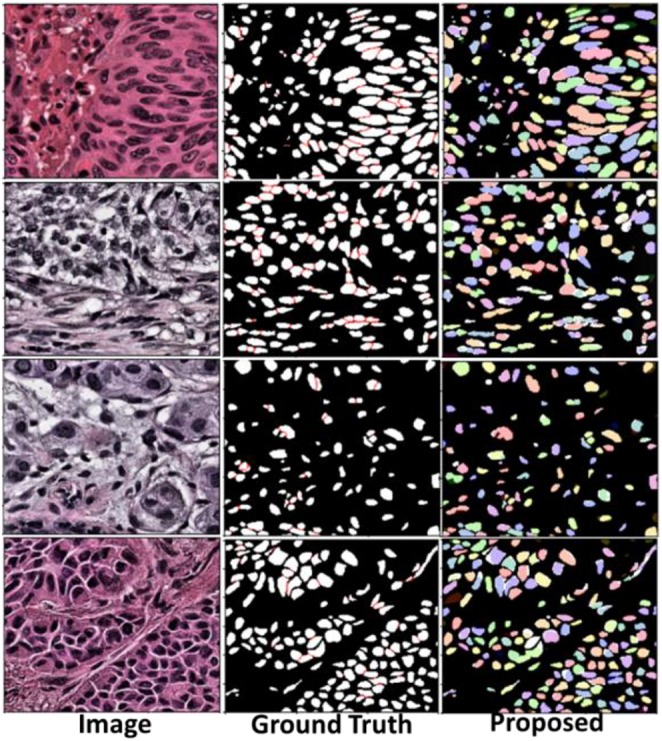
Segmentation result in validation dataset. The **left column** shows tissue images. The **middle column** is ground truth masks. The **right column** shows results from the segmentation method.

The Mask-RCNN model with ResNet-101 backbone obtained the 45.02% mean IOU (mIOU) on fivefold validation dataset. mIOU is the average precision score for each IOU with different thresholds (from 0.05 to 0.95 in the challenge). Tissue images with nuclei detections and segmentations are illustrated in Figure 10. A Dice score of 0.868 was achieved with the test dataset.

### Classification of Cancer Cases

On testing the algorithms on a dataset containing 20 radiology and pathology images, the three methods in Section “Methods for Classification of Brain Cancer Cases” achieved the accuracy scores (i.e., the number of correctly classified cases divided by the total number of cases) as shown in Table 1.

**TABLE 1:** Accuracy scores of the classification methods presented in Section “Methods for Classification of Brain Cancer Cases.”

**Method**	**Score**
Section “An Approach for Classification Of Low-Grade Gliomas Using Combined Radiology and Pathology Image Data”	0.90
Section “Dropout-Enabled Ensemble Learning for Multi-Scale Biomedical Image Classification”	0.80
Section “A Weighted Average-Based Classification Method”	0.75

## Discussion and Conclusion

Biomedical imaging has made great strides in image resolution and image capture speeds over the past decade. Radiology has enjoyed a widespread adoption for many years in both research and clinical settings. New imaging technologies are now allowing researchers to capture larger volumes of more detailed radiology data. Digital microscopy scanners were emerging technologies about 20 years ago. They required constant attention to capture sharp images of tissue and took many hours to scan a tissue specimen at moderate magnification levels. Nowadays, hundreds of slide tissues can be automatically imaged in several minutes. New scanning technologies and tissue staining methods are enabling researchers to capture richer morphological information at unprecedented resolutions. We anticipate that the FDA’s approval in 2017 of WSIs as a primary diagnostic tool will fuel a rapid increase in adoption of virtual slide technologies by researchers and clinicians. Combined with cheaper storage space, more powerful computing capabilities (via multi-core CPUs and accelerators such as graphics processing units), and Cloud computing infrastructures, biomedical imaging is rapidly becoming an essential tool in cancer research.

On the image analysis front, deep learning methods have seen a tremendous intake from the imaging community. These methods have demonstrated excellent results in the analysis of natural images. A rapidly growing collection of efforts are adapting these methods and extending them in innovative ways for application in biomedical image analysis. The segmentation method presented in this work shows the use of Mask-RCNN along with a non-maximum suppression (NMS) module for robust segmentation of nuclei in WSIs. The image classification methods employ a variety of deep learning methods and combine information from both radiology and pathology images to improve classification accuracy. All the methods described in this paper were evaluated with image ground truth data generated in the MICCAI CPM 2018 challenge (organized by a subset of the co-authors as denoted in the author list). The experimental results for nucleus segmentation show that high performance (i.e., high Dice scores) can be achieved by integrated use of Mask-RCNN and NMS for nucleus segmentation. The results for the classification methods show that a carefully assembled set of pipelines for each imaging modality and combination of prediction results from individual models can produce high classification accuracy.

While our work and works by other research teams have shown significant progress with more accurate, efficient, and robust image analysis algorithms, there remain challenges. One of the major challenges in machine learning analysis of biomedical imaging data is the lack of large curated and annotated training datasets, primarily because of time effort and domain expertise required for manual segmentations and classifications of tissue regions and micro-anatomic structures, such as nuclei and cells, as well as because of privacy and ownership concerns of source datasets. Some initial studies in the field of distributed learning in medicine attempted to address the data privacy and ownership challenge (Chang et al., 2018b; Sheller et al., 2018). These approaches need more investigation and adoption to facilitate collaboration across multiple medical institutions. Some projects have looked at the use of synthetic training datasets. Mahmood et al. (2018), for example, devised a method based on a conditional generative adversarial network (GAN) to improve deep learning-based segmentation of nuclei. Their method trains segmentation models using synthetic and real data. The authors employed a cycle GAN method to generate pairs of synthetic image patches and segmentation masks with varying amounts of touching and clumped nuclei. Such nuclei are difficult to segment by automated algorithms. In another work, Hou et al. (2019a) proposed a GAN architecture for the generation of synthetic tissue images and segmentation masks. The GAN architecture consists of multiple CNNs; a set of CNNs generates and refines synthetic images and masks to reference styles, and another CNN is trained online with these images and masks to generate a segmentation model. Another GAN approach was proposed by Senaras et al. (2018b) for tumor grading. The GAN network generates synthetic image datasets with known amounts of positive and negative nuclei in immunohistochemistry-stained tissue specimens (Senaras et al., 2018b).

Another major challenge in automated biomedical image analysis is the quality assessment of input datasets and analysis results. This also is a time-consuming and labor-intensive task, as automated algorithms can process large numbers of images and generate large volumes of analysis output to be reviewed and validated, thanks to advances in computing systems. There is a need to automate the quality assessment and validation processes. Some projects are looking at this problem. A recent work by Senaras et al. (2018a) used deep learning methods to detect out-of-focus regions in WSIs so that image analysis pipelines can avoid such regions. An approach proposed by Wen et al. utilized multiple machine learning methods, namely, SVM, random forest, and CNN, to assess the quality of nuclear segmentation results. The proposed approach made use of texture and intensity features extracted from image patches in a WSI to train the quality control models (Wen et al., 2017, 2018).

As our capability to capture complex radiology and pathology image data more rapidly and at higher resolutions evolves, manual training data generation and quality evaluation will become increasingly infeasible. We expect that (semi-)automated approaches, for training data generation, for assessing the quality of data and analysis results, and for iterative refinement of deep learning models, will become important tools in a researcher’s and clinician’s imaging toolset. We also believe image analysis challenges, such as the MICCAI 2018 CPM challenge, are important in efforts to develop more robust methods for image analysis and method assessment and validation. One of the issues that face machine/deep learning algorithm developers is the limited amount of ground truth datasets in biomedical imaging—the small dataset size is a limitation in our work as well. Thus, in addition to providing a platform for researchers to evaluate their methods in a controlled environment, image analysis challenge events contribute to a growing set of curated datasets that are valuable resources for development and refinement of future segmentation and classification algorithms. As part of our work, we make the datasets used in this challenge available to other researchers upon request.

## Data Availability Statement

The datasets analyzed for this study can be found in The Cancer Genome Atlas (https://portal.gdc.cancer.gov) and The Cancer Imaging Archive repositories (https://www.cancerimagingarchive.net). Please contact the corresponding author for details about the datasets and how to obtain them.

## Author Contributions

TK, SB, JK-C, JS, and KF were the main organizers of the MICCAI 2018 CPM satellite event presented in Section “Segmentation of Nuclei.” JD, TZ, RG, SB, and TK generated the datasets used in the challenge, supervised the collection of ground truth data, and developed the methods for evaluation of the challenge submissions. XR, LZ, QW, and DS proposed and implemented the nucleus segmentation algorithm presented in Section “Related Work.” YH, QQ, YZ, and XD proposed and implemented the classification algorithm described in Section “Datasets for Segmentation of Nuclei in Pathology Images.” AB, AsK, AvK, MK, and GK proposed and implemented the classification algorithm described in Section “Datasets for Combined Radiology and Pathology Classification.” All of the authors contributed text to the paper and edited it.

## Conflict of Interest

The authors declare that the research was conducted in the absence of any commercial or financial relationships that could be construed as a potential conflict of interest.

## Footnotes

1 https://portal.gdc.cancer.gov

2 https://www.cancerimagingarchive.net

3 https://www.cancer.gov/about-nci/organization/ccg/research/structural-genomics/tcga/using-tcga/types

4 https://github.com/matterport/Mask_RCNN
